# Convolutional Neural Networks to Classify Alzheimer’s Disease Severity Based on SPECT Images: A Comparative Study

**DOI:** 10.3390/jcm12062218

**Published:** 2023-03-13

**Authors:** Wei-Chih Lien, Chung-Hsing Yeh, Chun-Yang Chang, Chien-Hsiang Chang, Wei-Ming Wang, Chien-Hsu Chen, Yang-Cheng Lin

**Affiliations:** 1 Department of Physical Medicine and Rehabilitation, National Cheng Kung University Hospital, College of Medicine, National Cheng Kung University, Tainan 704, Taiwan; 2 Department of Physical Medicine and Rehabilitation, College of Medicine, National Cheng Kung University, Tainan 701, Taiwan; 3 Faculty of Information Technology, Monash University, Victoria 3800, Australia; 4 Department of Industrial Design, National Cheng Kung University, Tainan 701, Taiwan; 5 Department of Statistics, College of Management, National Cheng Kung University, Tainan 701, Taiwan

**Keywords:** convolutional neural network (CNN), Alzheimer’s disease (AD), single-photon emission computed tomography (SPECT), transfer learning, image recognition

## Abstract

Image recognition and neuroimaging are increasingly being used to understand the progression of Alzheimer’s disease (AD). However, image data from single-photon emission computed tomography (SPECT) are limited. Medical image analysis requires large, labeled training datasets. Therefore, studies have focused on overcoming this problem. In this study, the detection performance of five convolutional neural network (CNN) models (MobileNet V2 and NASNetMobile (lightweight models); VGG16, Inception V3, and ResNet (heavier weight models)) on medical images was compared to establish a classification model for epidemiological research. Brain scan image data were collected from 99 subjects, and 4711 images were used. Demographic data were compared using the chi-squared test and one-way analysis of variance with Bonferroni’s post hoc test. Accuracy and loss functions were used to evaluate the performance of CNN models. The cognitive abilities screening instrument and mini mental state exam scores of subjects with a clinical dementia rating (CDR) of 2 were considerably lower than those of subjects with a CDR of 1 or 0.5. This study analyzed the classification performance of various CNN models for medical images and proved the effectiveness of transfer learning in identifying the mild cognitive impairment, mild AD, and moderate AD scoring based on SPECT images.

## 1. Introduction

With a rapidly aging society, the number of people with Alzheimer’s disease (AD) is increasing globally. Approximately 9.9 million new cases of dementia are reported annually, which implies that a new patient is diagnosed with the disease every 3.2 s. The number of people with AD is expected to exceed 100 million by 2050. However, an effective medical treatment for the disease is yet to be devised. Neuroimaging has been used for understanding the progression of AD, and considerable progress has been achieved in the use of deep learning for medical imaging in research and clinical medicine [1]. The macroscopic findings of AD have revealed diffuse brain atrophy [2]. The classification accuracy of AD versus healthy control using deep learning of magnetic resonance imaging (MRI) is 91.4%, and that of mild cognitive impairment (MCI) versus AD is 70.1% [3].

The economic evaluation of dynamic susceptibility MRI compared with nonenhanced computer-assisted tomography (CT) is USD 479,500 per quality-adjusted life year, whereas the corresponding comparison for single-photon emission computed tomography (SPECT) with CT is better (higher effectiveness and lower cost) [4,5]. Regional cerebral blood flow (rCBF) is related to brain metabolism; therefore, changes in CBF reflect variations in neuronal metabolism. From the perspective of neuropathology, subjects with very mild AD typically exhibit abnormal metabolic and rCBF patterns, even at the preclinical stage [6]. A decreased rCBF already occurs in individuals with MCI before they transition to AD [7]. A disrupted cerebral perfusion may cause impaired vascular clearance ability, which promotes the deposition of beta-amyloid and neurofibrillary tangles. Clinical studies have revealed that rCBF alterations are involved in AD pathogenesis. Even before the accumulation of beta-amyloid, subjects with high risk exhibit changes in cerebral blood flow [8]. In addition to clinical manifestations, rCBF SPECT, such as voxel-as-features, has frequently been used by physicians as a diagnostic tool [9,10].

Unlike general natural image recognition tasks, large, labeled training datasets are yet to be devised for medical image analysis [11]. These inadequate labeled data for supervised machine learning using electronic health records are the primary bottleneck in the model development [12]. Therefore, many models based on transfer learning-based methods have been proposed to address this concern [13]. In transfer learning, a previously trained model is applied to another field to improve the learning method for a few labels or small number of datasets in the target data. Pretrained convolutional neural networks (CNNs) (OverFeat) have been used in transfer learning to identify and detect vertical pathologies using X-ray and CT modalities [14]. In previous studies, the accuracy of pretrained CNNs using MRI to detect AD was 0.40 [15]. A fully convolutional network has been used with transfer learning for identifying malignant breast lesions [16] and retinal blood vessel segmentation [17]. Overfitting can easily occur when a small dataset is directly used to train deep learning networks. Transfer learning can improve the initial ability to extract features to alleviate this risk [13]. In this case, transfer learning between task domains is desirable. Furthermore, a fine-tuned CNN after transfer learning should always be the preferred option, regardless of the size of the available training sets. Additionally, the fine-tuned CNN model after transfer learning can quickly attain the maximum performance [18]. By contrast, CNNs trained from scratch require extensive training to achieve the highest performance. Therefore, transfer learning was used in this study to conduct experiments. Such a method can be executed even with limited training data.

In previous studies, rCBF SPECT for the diagnosis of AD revealed a sensitivity of 86%, specificity of 73%, and accuracy of 82% [19]. Brain perfusion SPECT has been proven to be a sensitive tool for assessing functional deficits in the early stages of AD [20]. MCI, referred to with a clinical dementia rating (CDR) of 0.5, with a pooled sensitivity and specificity of 93% and 97%, respectively [21], is an emerging tool for early detection and intervention. A study [9] used voxel-as-features with k-nearest neighbor classification to develop a set of diagnostic models for SPECT imaging. To highlight the benefits of the proposed approach in the early diagnosis of AD, 180 SPECT images with Tc-99m ethyl cysteinate dimer (ECD) as the tracer were used, including 43 normal participants and 30 participants with possible AD. The accuracy of classification of patients with possible AD and normal controls was 71.67%, which indicated acceptable accuracy of the conventional machine learning method. To the best of our knowledge, rCBF SPECT has not been used to classify MCI and mild and moderate AD. Specifically, few studies have comprehensively analyzed the various types of CNN models and discussed their applications in medical imaging [13,22,23].

This study compared various methods based on CNN models for the detection and medical diagnosis of MCI and AD. Specifically, the detection performances of the lightweight CNN models MobileNet V2 [24] and NASNetMobile [25] and the heavier weight CNN models VGG16 [26], Inception V3 [27], and ResNet [28] were compared. These five CNN models are the most widely used in transfer learning for disease diagnosis using medical imaging [29]. The findings of this study can assist physicians in the early stages of clinical diagnosis and reduce the occurrence of misdiagnoses.

## 2. Materials and Methods

This study was approved by the National Cheng Kung University Human Research Ethics Committee (NCKU HREC-E-108-282-2) and was conducted in accordance with the principles of the Declaration of Helsinki. All participants provided written informed consent. The experimental steps for training the CNN model were divided into two stages. This study compared the accuracies of various architectures of CNN models in predicting SPECT images. In terms of the analysis strategy, adjustments were made to three influencing factors, namely optimizer, fully connected layers, and model parameters, to optimize the CNN model performance. The two-stage experimental process is illustrated in Figure S1.

### 2.1. Participants

Inclusion criteria: (1) Patients who were over 60 years and sought evaluation for cognitive function decline in the Department of Neurology, National Cheng Kung University Hospital from January 2017 to December 2019. (2) Patients who completed comprehensive cognitive function tests, with results including mini mental state exam (MMSE) score, cognitive abilities screening instrument (CASI) score, CDR score, and sum of box (SOB) and brain SPECT imaging data with Tc-99m ECD as a tracer. Clinical history, physical and neurological examinations, laboratory, and instrumental investigations (including routine laboratory tests, thyroid hormone status, vitamin B12, folate levels, EEG, and CT or MRI brain scans) were performed to exclude secondary dementia. Experienced neurologists evaluated these image data for the possibility of AD and the detailed diagnosis, including the education level, the MMSE score, CASI score, CDR score, and SOB. Brain SPECT image data were collected from 99 subjects, 36 men and 63 women. Detailed demographic and diagnostic data are presented in Table 1.

### 2.2. SPECT Image Dataset

**Figure 1:**
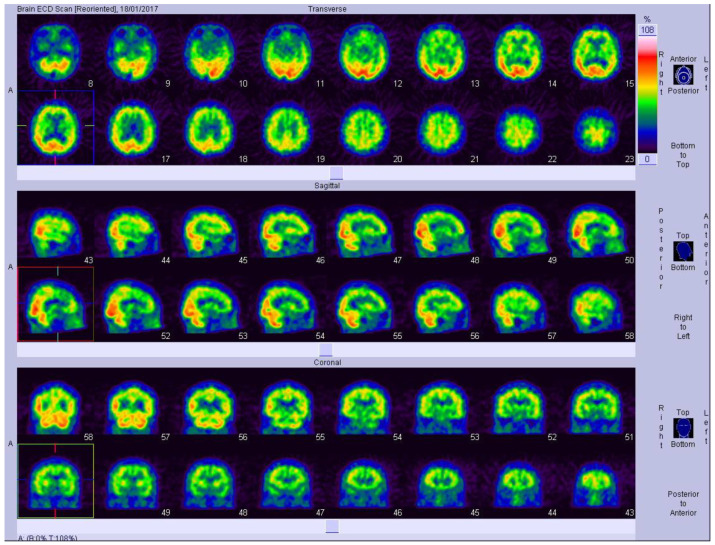
Single-photon emission computed tomography (SPECT) images of one participant. A total of 16 SPECT images were obtained in one section of one patient. Next, 48 SPECT images were obtained in three sections of this participant. A total of 4752 (48 × 99) SPECT images of all 99 participants were used. Of the 4752 images, 41 images beyond the area of brain were deleted. Therefore, 4711 images were used for further analysis. The colors in the figure represent the ranges of blood flow with red being the most blood flow and violet the least.

**Figure 2:**
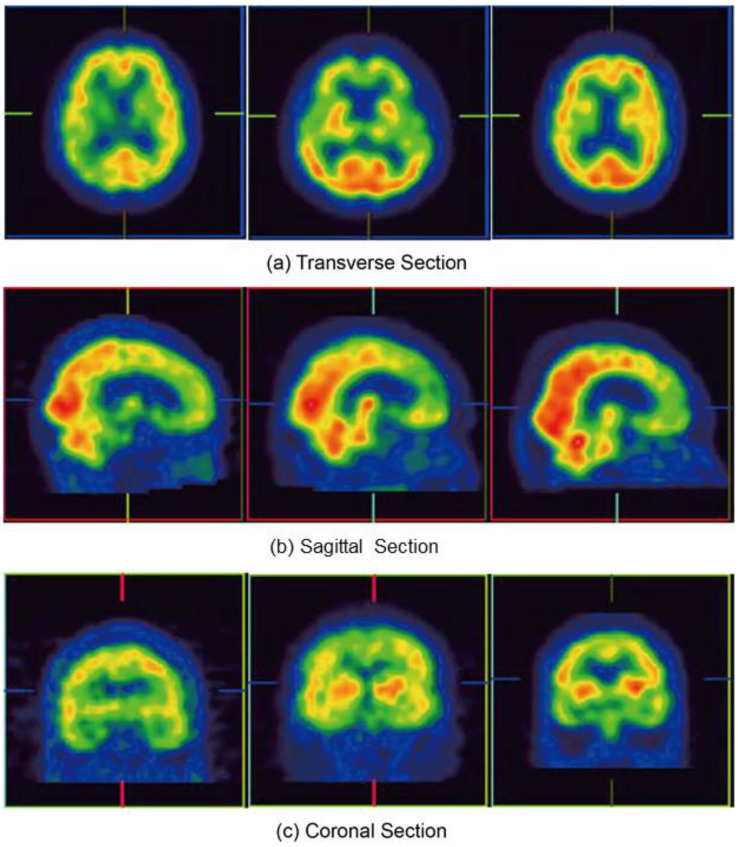
Three sections of SPECT images. (**a**) Transverse section; (**b**) Sagittal Section; (**c**) Coronal section. The colors in the figure represent the ranges of blood flow with red being the most blood flow and violet the least.

Generally, the collection of image data suitable for developing a brain imaging recognition system is one of the hardest steps in the procedure. In this study, SPECT imaging data with Tc-99m ECD as a tracer were used to test the effectiveness of the proposed method. To avoid deviations between different hospital sources, the images used were obtained from a single hospital; specifically, the archives of the Department of Neurology, National Cheng Kung University Hospital (Figure 1 and Figure 2).

**Figure 3:**
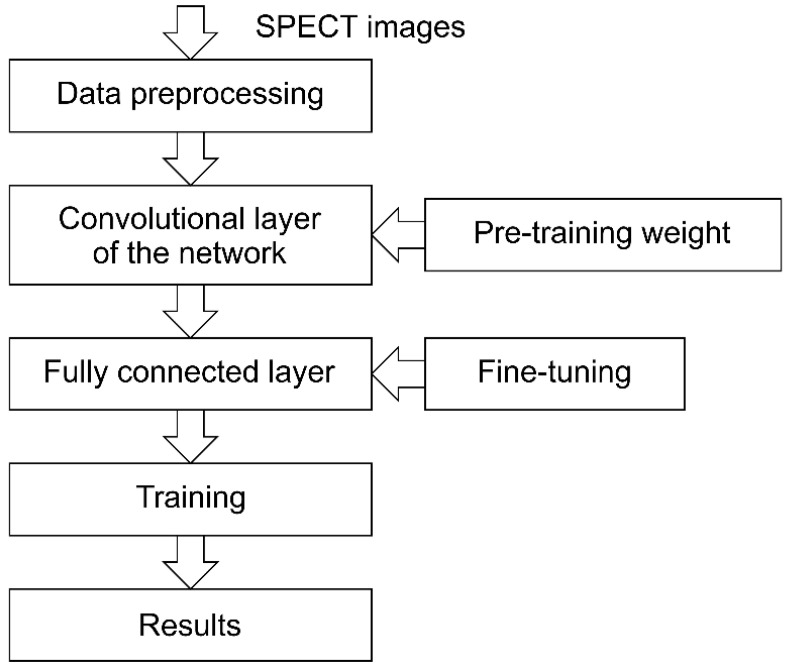
Process and architecture of CNN models.

To reduce unnecessary information in the images and reduce model training errors, images of all 99 participants were cropped to remove excess background information [30]. After the preprocessing operation, 4711 images of individual slices (as displayed in Figure 3) were obtained. Each cropped SPECT image was a JPG file with a size of 124 × 120 pixels. Of the 99 patients, 52 patients were categorized as having questionable dementia (CDR = 0.5), 39 had MCI (CDR = 1), and 8 had moderate cognitive impairment (CDR = 2). The 4711 images were classified into three categories based on the CDR: 4461 were used as training data (80%) and verification data (20%) to perform all the experiments, and the remainder were manually used as test data (250 images) as suggested in previous studies [31].

### 2.3. Architecture of Five CNN Models after Transfer Learning

After obtaining the SPECT images (Figure 1), the voxel intensities were directly used as features. In the feature set, graphics were output in the transverse/sagittal/coronal sections of three intelligent identifications. In machine learning classification, a neural network was used to classify the three feature sets of CDR 0.5, 1, and 2. The neural network-like architecture is displayed in Figure 3, in the order of the graphics input layer (the size is 124 × 120 × 3), convolution layer, pooling layer, and fully connected layer. The fully connected layer groups and classifies the features extracted by the previous convolutional layer. Furthermore, to verify the accuracy of the prediction, each image is analyzed through an activation function and an output probability value is obtained (Figure 3 and Table 2).

This study used transfer learning to load the weights of the pretrained model on the new network structure and then trained the network to recognize SPECT images. Five CNNs, namely MobileNet V2, NASNetMobile, VGG16, Inception V3, and ResNet, were used. The structure of the pretrained model was modified through fine-tuning and was then used as the initial model for the SPECT image recognition task. First, we preprocessed the SPECT image data and then used the original convolutional layer in the network structure to extract bottleneck features. Second, we connected the fine-tuned fully connected layer to form a new network structure. The experimental process is illustrated in Figure 3. The fine-tuning process of the fully connected layers in various CNN models is presented in the following subsections. The details of the two lightweight CNN models, namely, MobileNet V2 and NASNetMobile, are presented in the Supplementary Materials.

### 2.4. VGG16

#### 2.4.1. Fine-Tuning

First, a flattened layer was added to dimensionalize the output of the previous convolutional layer into a two-dimensional matrix. This method reduced the image size without affecting important image features. The output dimension of DenseNet was set to 1024. Finally, the dropout layer was added, and the ratio was set to 0.5 to improve model generalization and avoid excessive reliance on certain regional features (see Table 2).

#### 2.4.2. Experimental Setup

The dataset was normalized to improve data integrity and ensure the similarity of appearance and reading methods of all image data in the records. SoftMax was used as the resulting classifier. For VGG16, the experimental settings (ADAM optimization method, learning rate, exponential decay rate, and attenuation value) were the same as those of MobileNet V2. ReLU was used as the activation function. To increase the amount of training data, we performed rotation, shearing strength, horizontal flip, random scaling, filling, and other processes on the original image (see Table 2).

### 2.5. Inception V3

#### 2.5.1. Fine-Tuning

To prevent excessive model parameters from causing overfitting problems, Global Average Pooling 2D was added at the end of the model to replace the fully connected layer, and the average value of each feature map was selected as the output. Subsequently, a dense layer was added, and the output dimension was set to 2048 (see Table 2 for details).

#### 2.5.2. Experimental Setup

The input image size of Inception V3 is 299 × 299 pixels. Therefore, rescale = 1/255 was added in the preprocessing stage of the image data, and each pixel value of the SPECT image with an original size of 124 × 120 pixels was multiplied by the scaling factor to facilitate model convergence. To present the classification results as percentages, SoftMax was added as the resulting classifier, and the classification category was set to 3. Similar to the NASNetMobile model, SGD was selected as the optimizer of the model to increase the training speed. The learning rate was set to 10^−5^, momentum was 0.9, loss function was categorical cross-entropy, batch size was 64, and epoch number was 50. Table 2 details the horizontal flip, random zoom, and original image for data enhancement.

### 2.6. ResNet

#### 2.6.1. Fine-Tuning

**Figure 4:**
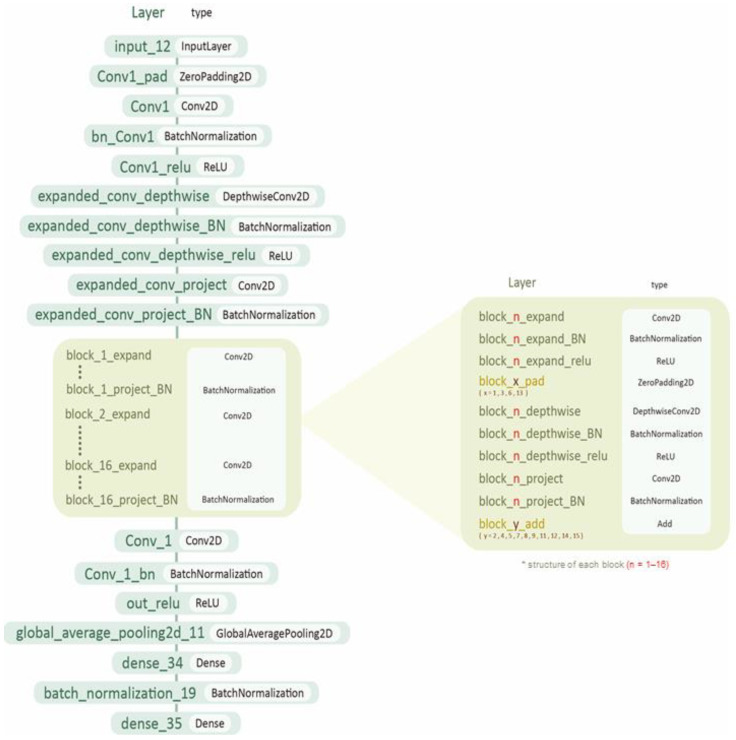
Architecture of the ResNet model.

The flattened layer was added to dimensionalize the output of the previous convolutional layer into a two-dimensional matrix, and batch normalization was added. A dense layer was included, and the output dimension was set to 256. Finally, the dropout layer was set to 0.5 to avoid too much reliance on certain regional features. Table 2 details layers added at the end of the ResNet after fine-tuning. Figure 4 illustrates the architecture of the ResNet model.

#### 2.6.2. Experimental Setup

As described for NASNetMobile, rescale = 1/255 was added in the preprocessing stage of the image data to facilitate model convergence. To present the classification results as percentages, SoftMax was selected as the result classifier and SGD was the optimization method. The learning rate was set to 10^−4^, momentum was 0.9, loss function was categorical cross-entropy, batch size was set to 32, epoch number was set to 50, and activation function was ReLU. Before data analysis, the data enhancement method was used to perform position shift, horizontal flip, and fill processing on the original image (Table 2).

### 2.7. Statistical Analysis

All 4711 image data were randomly categorized into three separate data frames, namely training, validation, and test sets. First, 250 image data were manually extracted as test data. Subsequently, the random train–validation split was used to divide all remaining data into training (80%) and validation (20%) datasets. The training dataset was used to fit the model, and the validation dataset was used to validate the generalization ability of the model during the training process. The validation and training datasets remained unchanged to avoid the training–serving skew. The demographic data were expressed as the mean ± standard deviation and compared using the chi-squared test and one-way analysis of variance with Bonferroni’s post hoc test. The performance of CNN models was evaluated using the learning curves of model accuracy and loss [32]. Learning curves are widely used in machine learning for models that optimize their internal parameters incrementally over training cycles (epochs). The metric used to evaluate learning could be maximizing, which revealed that better scores of classification accuracy indicate more learning. Using a score that is minimizing, such as loss, is preferable, where better scores (smaller loss) indicate more learning, and a value of 0 indicates that the training dataset was learned perfectly, and no errors were made. The confusion matrix was also calculated. A *p* value of <0.05 was considered statistically significant.

## 3. Results

Brain scan image data were collected from 36 men and 63 women. The demographic characteristics of the subjects are presented in Table 1.

Subjects with CDRs of 2 were significantly older than those with CDRs of 0.5. The SOB scores of subjects with CDR of 2 were significantly higher than those of subjects with CDRs of 1 and 0.5. The CASI and MMSE scores of subjects with CDR of 2 were significantly lower than those of subjects with CDRs of 1 and 0.5 (Table 1). The scores of the SOB, CASI, and MMSE validated the severity of cognitive impairments classified using the CDR score.

Following the evaluation of the severity of cognitive impairments, SPECT images were analyzed using two lightweight and three heavier weight CNN models to distinguish the severity of AD in patients based on the CDR scores. We used two evaluation indicators, namely accuracy and loss, to evaluate the detection performance of the model.

We first examined the effect of the various sections of brain images (i.e., the transverse, sagittal, and coronal sections) on the model to improve its performance in detecting the severity of AD by detecting different sections of SPECT images. A total of 1602 transverse images, 1584 sagittal images, and 1525 coronal images were obtained. Subsequently, the SPECT image data of the three cross-sections were mixed for model identification experiments.

Table 3 lists the performance indicators of the five CNN models that identify different CDR scores from a single brain section and a mixed section in the validation and testing data. Based on the classification results for a single brain section presented in Table 3, the ResNet model was the best performer among the five CNN models. The validation accuracy rates in the transverse, sagittal, and coronal section image data were 67.23%, 65.37%, and 68.51%, respectively. Inception V3 was the worst-performing CNN model with validation accuracy rates of 56.77%, 53.09%, and 52.78%. The classification validation accuracy of MobileNet V2, NASNetMobile, and VGG16 was approximately 60–69%.

For the classification results of the mixed section, Table 3 reveals that the best model was ResNet, with a validation accuracy rate of 72.39% and a test accuracy rate of 68.8% (confusion matrix in Figure S2; the precision, recall, and F1 score for each class of CDR scores in Table S1), which is sufficient for determining the performance of various CNN models for medical images. The second highest accuracy rate was 69.45% (VGG16). Inception V3 was the worst-performing model, with a validation accuracy rate of only 56.77%. Based on the experimental results, the lightweight CNN models (MobileNet V2 and NASNetMobile) were unsatisfactory. For the image data obtained by mixing the three sections, the best validation accuracies of MobileNet V2 and NASNetMobile were only 61.87% and 59.89%, respectively, indicating that the two lightweight CNN models can be improved to match SPECT medical images. The epoch of each CNN model after transfer learning was set to 50, and the learning curves of model accuracy and loss of mixed data using each CNN model are displayed in Figures S3–S6. The loss of the ResNet model after transfer learning was the smallest, which indicated that it learned the most (Figure S7). The values of validation accuracy were higher than those of training accuracy in the NASNetMobile, VGG16, Inception V3, and ResNet models after approximately 40 epochs. This phenomenon indicated that validation improved the classification accuracy in these four models after a longer training duration (Figures S4–S6).

## 4. Discussion

Neuroimaging has become a useful tool for understanding AD pathogenesis, and the use of deep learning techniques in medical imaging has achieved considerable progress in research and clinical care.

In this study, five CNN models were trained in the transfer learning process, and performance evaluations and comparisons were performed. The primary objective of this study was to compare the detection performance of various CNN structures for medical images, which was confirmed based on the results. Moreover, compared with using a single cross-sectional image as the input, the use of mixed data from the three cross-sections as the model input produced excellent results.

**Figure 5:**
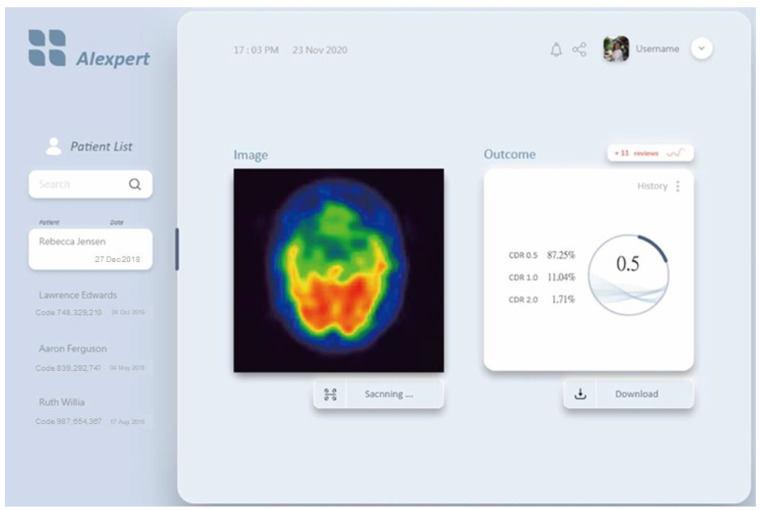
AI expert system based on the ResNet model. Physicians can import the SPECT image (left part), and the AI expert system will show the outcome (here, of 0.5) (right part) to help them make decisions on disease classification.

The validation and test accuracy records in Table 3 indicate that the performance of ResNet was superior to those of MobileNet V2, NASNetMobile, VGG16, and Inception V3. In this type of medical imaging dataset, the heavier weight networks performed better than the lightweight networks. Furthermore, ResNet can effectively avoid gradient explosion, disappearance, and network degradation. Therefore, ResNet can be used to develop an AI expert system that can analyze AD severity using SPECT images, as displayed in Figure 5. The AI expert system does not replace physicians but helps them achieve clearer decisions on disease classification and more confident diagnosis based on systematic objective information to reduce the uncertainty in disease classification diagnosis.

The performance of CNN models, such as ResNet, varies depending on the type of study, field of study, data used, and imbalance in the sample [33]. In neuroimaging research, image processing and feature recognition have been applied to AD classification. Among the various deep learning methods, ResNet has been widely used for the classification and diagnosis of AD. Amin-Naji et al. [34] used a residual structure in each branch of a CNN. The OASIS dataset was used to evaluate the effectiveness of the model. Finally, an accuracy of 98.72% was obtained in the classification of old patients with AD and normal controls using MRI, which is the best result compared with those in other previous studies on the same database.

Abrol et al. [35] evaluated the ability of a residual structure to learn from structural MRI data using neuroimaging and revealed that it is binary classification or heap classification. Their results showed that the recognition performance of the ResNet architecture is better than that of the SVM and SAE methods. Karasawa and Ohwada [36] proposed a ResNet-based structure for classifying MRI data from the Alzheimer’s Disease Neuroimaging Initiative database. The experimental results indicated that the proposed 39-layer residual structure had the highest accuracy compared with those of VGGNet and ResNet-50. Therefore, related research has revealed that the residual structure exhibited a certain effect on the applicability of image recognition in neuroimaging. This finding revealed that the ResNet structure used in this study is effective for AD classification. In previous studies, the performance of the total and subscale scores of the Mattis Dementia Rating Scale for discriminating MCI from NC, MCI from mild AD, and mild AD from moderate AD revealed an accuracy from 61% to 85% [37], which was comparable with the performance of our rCBF SPECT CNN model.

## 5. Conclusions

Although this study revealed that deep learning technology can achieve excellent performance in SPECT image classification, performance can be improved. The proposed method provides important implications for image recognition and deep learning in the development of mobile applications in AI and medical treatment and exhibits considerable potential in other biomedical fields. The proposed method may open novel avenues for medical image analysis and provide a potentially accurate CNN architecture for researchers and physicians to predict new data. In the future, we hope to collect more clinical data from various hospitals to increase the depth of the training dataset. Moreover, neuroimaging information can be combined with cognitive scale functional information to obtain a superior machine learning model for the improved classification of AD severity.

## Figures and Tables

**Table 1:** Subjects’ demographic and clinical information.

	CDR (0.5)	CDR (1)	CDR (2)	*p* Value
N	52	39	8	
Age (years)	72.08 ± 7.96	76.15 ± 8.91	80.11 ± 3.22	0.01 * (CDR (0.5) < CDR (2))
Gender (female/male)	27/25	31/8	5/3	0.03 *
Education (years)	8.43 ± 4.40	7.03 ± 4.13	7.00 ± 6.08	n.s.
SOB	1.30 ± 0.72	4.73 ± 1.31	10.39 ± 0.93	<0.01 ** (CDR (0.5) < CDR (1); CDR (1) < CDR (2))
CASI	76.96 ± 11.80	63.26 ± 13.68	48.89 ± 14.91	<0.01 ** (CDR (0.5) > CDR (1); CDR (1) > CDR (2))
MMSE	22.92 ± 4.04	19.18 ± 3.91	14.78 ± 3.96	<0.01 ** (CDR (0.5) > CDR (1); CDR (1) > CDR (2))

Note: Values are numbers or mean ± standard deviation (range). Abbreviations: CDR = Clinical Dementia Rating Scale; MMSE = Mini Mental State Exam; N = Number; CASI = Cognitive Abilities Screening Instrument; SOB = Sum of Box. Data were compared using the chi-squared test and one-way analysis of variance with Bonferroni’s post hoc test. * *p* < 0.05, ** *p* < 0.01, *p* > 0.05; no significant difference (n.s.).

**Table 2:** Layer settings after transfer learning and preprocessing for data enhancement.

**(a) MobileNet V2**	Layer	Type
Layer Setting	global_average_pooling2d_1	GlobalAveragePooling2D
	batch_normalization_1	BatchNormalization
	dense_1	Dense
	batch_normalization_2	BatchNormalization
	dropout_1	Dropout
Data Enhancement	height_shift_range = 0.2	
	width_shift_range = 0.2	
	shear_range = 0.2	
	zoom_range = 0.2	
	horizontal_flip = True	
	fill_mode = nearest	
**(b) NASNetMobile**	Layer	Type
Layer Setting	global_average_pooling2d_1	GlobalAveragePooling2D
	dense_1	Dense
	dropout_1	Dropout
Data Enhancement	height_shift_range = 0.2	
	shear_range = 0.2	
	horizontal_flip = True	
	fill_mode = nearest	
**(c) VGG16**	Layer	Type
Layer Setting	flatten	Flatten
	dense_1	Dense
	dropout_1	Dropout
	dense_2	Dense
Data Enhancement	rotation_range = 40	
	height_shift_range = 0.2	
	shear_range = 0.2	
	zoom_range = 0.2	
	horizontal_flip = True	
	fill_mode = nearest	
**(d) Inception V3**	Layer	Type
Layer Setting	global_average_pooling2d_1	GlobalAveragePooling2D
	dense_1	Dense
Data Enhancement	height_shift_range = 0.2	
	horizontal_flip = True	
	fill_mode = nearest	
**(e) ResNet**	Layer	Type
Layer Setting	flatten	Flatten
	batch_normalization_1	BatchNormalization
	dense_1	Dense
	dropout_1	Dropout
	dense_2	Dense
Data Enhancement	height_shift_range = 0.2	
	width_shift_range = 0.2	
	horizontal_flip = True	
	fill_mode = nearest	

**Table 3:** Detection performance of the two lightweight and three heavier weight CNN models.

Model	Types of Data	Validation Accuracy (%)	Test Accuracy (%)
MobileNet V2	Transverse section	60.57	60.01
	Sagittal section	58.69	50.45
	Coronal section	60.89	57.77
	Mixed (three kinds of section)	61.87	58.43
NASNetMobile	Transverse section	63.87	61.25
	Sagittal section	57.12	57.03
	Coronal section	63.03	55.49
	Mixed (three kinds of section)	59.89	58.77
VGG16	Transverse section	66.03	64.58
	Sagittal section	64.20	61.89
	Coronal section	58.66	53.01
	Mixed (three kinds of section)	69.45	67.53
Inception V3	Transverse section	56.77	54.22
	Sagittal section	53.09	50.98
	Coronal section	52.78	47.55
	Mixed (three kinds of section)	54.03	52.13
ResNet	Transverse section	67.23	61.20
	Sagittal section	65.37	63.37
	Coronal section	68.51	65.28
	Mixed (three kinds of section)	72.39	68.80

## Data Availability

The data presented in this study are available on request from the corresponding author. The data are not publicly available due to privacy concerns of research participants.

## Supplementary Materials

The following supporting information can be downloaded at: https://www.mdpi.com/article/10.3390/jcm12062218/s1, Table S1: Precision, recall, and F1 score of the ResNet model using the mixed test data of the SPECT brain images for each class of CDR scores; Figure S1: Two-stage experimental process utilized in this study; Figure S2: Confusion matrix of the ResNet model using the mixed test data of the SPECT brain images. 0.5: Clinical Dementia Rating Scale (CDR) = 0.5; 1: CDR = 1; 2: CDR = 2.; Figure S3: Learning curves of model accuracy and loss of mixed data using the MobileNetV2 model. The epoch of the MobileNetV2 model after transfer learning was set to 50, and the SPECT brain images were classified; Figure S4: Learning curves of model accuracy and loss of mixed data using the NasNetMobile model. The epoch of the NasNetMobile model after transfer learning was set to 50 and SPECT brain images were classified; Figure S5: Learning curves of model accuracy and loss of mixed data using the VGG 16 model. The epoch of the VGG 16 model after transfer learning was set to 50 and the SPECT brain images were classified; Figure S6: Learning curves of the model accuracy and loss of mixed data using the Inception V3 model. The epoch of the Inception V3 model after transfer learning was set to 50 and the SPECT brain images were classified; Figure S7: Learning curves of the model accuracy and loss of mixed data using the ResNet model. The epoch of the ResNet model after transfer learning was set to 50 and the SPECT brain images were classified.

## References

[1] Chetelat G.A., Baron J.-C. (2003). Early diagnosis of Alzheimer’s disease: Contribution of structural neuroimaging. Neuroimage.

[2] Hashizume Y. (2022). Macroscopic findings of brain with dementia. Neuropathology.

[3] Li F., Tran L., Thung K.H., Ji S., Shen D., Li J. (2015). A robust deep model for improved classification of AD/MCI patients. IEEE J. Biomed. Health Inform..

[4] Leung G.M., Yeung R.Y., Chi I., Chu L.W. (2003). The economics of Alzheimer disease. Dement. Geriatr. Cogn. Disord..

[5] Lien W.-C., Wang W.-M., Wang F., Wang J.-D. (2021). Savings of loss-of-life expectancy and lifetime medical costs from prevention of spinal cord injuries: Analysis of nationwide data followed for 17 years. Inj. Prev..

[6] Binnewijzend M.A.A., Benedictus M.R., Kuijer J.P.A., van der Flier W.M., Teunissen C.E., Prins N.D., Wattjes M.P., van Berckel B.N.M., Scheltens P., Barkhof F. (2016). Cerebral perfusion in the predementia stages of Alzheimer’s disease. Eur. Radiol..

[7] Binnewijzend M.A., Kuijer J.P.A., Benedictus M.R., van der Flier W.M., Wink A.M., Wattjes M.P., van Berckel B.N.M., Scheltens P., Barkhof F. (2013). Cerebral blood flow measured with 3D pseudocontinuous arterial spin-labeling MR imaging in Alzheimer disease and mild cognitive impairment: A marker for disease severity. Radiology.

[8] Chao L.L., Buckley S.T., Kornak J., Schuff N., Madison C., Yaffe K., Miller B.L., Kramer J.H., Weiner M.W. (2010). ASL perfusion MRI predicts cognitive decline and conversion from MCI to dementia. Alzheimer Dis. Assoc. Disord..

[9] Górriz J.M., Segovia F., Ramírez J., Lassl A., Salas-Gonzalez D. (2011). GMM based SPECT image classification for the diagnosis of Alzheimer’s disease. Appl. Soft Comput..

[10] Lien W.-C., Wang W.-M., Wang H.-M.D., Lin F.-H., Yao F.-Z. (2021). Environmental barriers and functional outcomes in patients with schizophrenia in Taiwan: The capacity-performance discrepancy. Int. J. Environ. Res. Public Health..

[11] Litjens G., Kooi T., Bejnordi B.E., Setio A.A.A., Ciompi F., Ghafoorian M., van der Laak J.A.W.M., van Ginneken B., Sánchez C.I. (2017). A survey on deep learning in medical image analysis. Med. Image Anal..

[12] Lien W.-C., Ching C.T.-S., Lai Z.-W., Wang H.-M.D., Lin J.-S., Huang Y.-C., Lin F.-H., Wang W.-F. (2022). Intelligent fall-risk assessment based on gait stability and symmetry among older adults using tri-axial accelerometry. Front. Bioeng. Biotechnol..

[13] Raghu M., Zhang C., Kleinberg J., Bengio S. (2019). Transfusion: Understanding transfer learning for medical imaging. Adv. Neural Inf. Process. Syst..

[14] van Ginneken B., Setio A.A., Jacobs C., Ciompi F. Off-the-shelf convolutional neural network features for pulmonary nodule detection in computed tomography scans. Proceedings of the 2015 IEEE 12th International Symposium on Biomedical Imaging ISBI.

[15] Simon B.C., Baskar D., Jayanthi V.S. Alzheimer’s disease classification using deep convolutional neural network. Proceedings of the 2019 9th International Conference on Advances in Computing and Communication (ICACC).

[16] Sun Y., Ma S., Sun S., Liu P., Zhang L., Ouyang J., Ni X. (2021). Partial discharge pattern recognition of transformers based on MobileNets convolutional neural network. Appl. Sci..

[17] Jiang Z., Zhang H., Wang Y., Ko S.B. (2018). Retinal blood vessel segmentation using fully convolutional network with transfer learning. Comput. Med. Imaging. Graph..

[18] Shin H.-C., Roth H.R., Gao M., Lu L., Xu Z., Nogues I., Yao J., Mollura D., Summers R.M. (2016). Deep convolutional neural networks for computer-aided detection: CNN architectures, dataset characteristics and transfer learning. IEEE Trans. Med. Imaging.

[19] Bonte F.J., Weiner M.F., Bigio E.H., White C.L. (1997). 3rd. Brain blood flow in the dementias: SPECT with histopathologic correlation in 54 patients. Radiology.

[20] Arnáiz E., Almkvist O. (2003). Neuropsychological features of mild cognitive impairment and preclinical Alzheimer’s disease. Acta Neurol. Scand..

[21] Huang H.-C., Tseng Y.-M., Chen Y.-C., Chen P.-Y., Chiu H.-Y. (2021). Diagnostic accuracy of the Clinical Dementia Rating scale for detecting mild cognitive impairment and dementia: A bivariate meta-analysis. Int. J. Geriatr. Psychiatry.

[22] Gajbhiye G.O., Nandedkar A.V., Faye I. (2022). Translating medical image to radiological report: Adaptive multilevel multi-attention approach. Comput. Methods Programs Biomed..

[23] Huynh B.Q., Li H., Giger M.L. (2016). Digital mammographic tumor classification using transfer learning from deep convolutional neural networks. J. Med. Imaging.

[24] Sandler M., Howard A., Zhu M., Zhmoginov A., Chen L.C. Mobilenetv2: Inverted residuals and linear bottlenecks. Proceedings of the IEEE Conference on Computer Vision and Pattern Recognition.

[25] Zoph B., Vasudevan V., Shlens J., Le Q.V. Learning transferable architectures for scalable image recognition. Proceedings of the IEEE Conference on Computer Vision and Pattern Recognition.

[26] Simonyan K., Zisserman A. (2014). Very Very deep convolutional networks for large-scale image recognition. arXiv.

[27] Szegedy C., Vanhoucke V., Ioffe S., Shlens J., Wojna Z. Rethinking the inception architecture for computer vision. Proceedings of the IEEE Conference on Computer Vision and Pattern Recognition.

[28] Targ S., Almeida D., Lyman K. (2016). ResNet in ResNet: Generalizing residual architectures. arXiv.

[29] Yu X., Wang J., Hong Q.-Q., Teku R., Wang S.-H., Zhang Y.-D. (2022). Transfer learning for medical images analyses: A survey. Neurocomputing.

[30] Lien W.-C., Zhou X.-R., Liang Y.-J., Ching C.T.-S., Wang C.-Y., Lu F.-I., Chang H.-C., Lin F.-H., Wang H.-M.D. (2023). Therapeutic potential of nanoceria pretreatment in preventing the development of urological chronic pelvic pain syndrome: Immunomodulation via reactive oxygen species scavenging and SerpinB2 downregulation. Bioeng. Transl. Med..

[31] Chen H., Zhang K., Lyu P., Li H., Zhang L., Wu J., Lee C.-H. (2019). A deep learning approach to automatic teeth detection and numbering based on object detection in dental periapical films. Sci. Rep..

[32] de Bruijne M. (2016). Machine learning approaches in medical image analysis: From detection to diagnosis. Med. Image Anal..

[33] Johnson J.M., Khoshgoftaar T.M. (2019). Survey on deep learning with class imbalance. J. Big Data.

[34] Amin-Naji M., Mahdavinataj H., Aghagolzadeh A. Alzheimer’s disease diagnosis from structural MRI using Siamese convolutional neural network. Proceedings of the 2019 4th International Conference on Pattern Recognition and Image Analysis IPRIA.

[35] Abrol A., Bhattarai M., Fedorov A., Du Y., Plis S., Calhoun V., for the Alzheimer’s Disease Neuroimaging Initiative (2020). Deep residual learning for neuroimaging: An application to predict progression to Alzheimer’s disease. J. Neurosci. Methods.

[36] Karasawa H., Liu C.-L., Ohwada H. (2018). Deep 3D convolutional neural network architectures for Alzheimer’s disease diagnosis. Intelligent Information and Database Systems.

[37] Qian S., Chen K., Guan Q., Guo Q. (2021). Performance of Mattis dementia rating scale-Chinese version in patients with mild cognitive impairment and Alzheimer’s disease. BMC Neurol..

