# Supplementary material for: Convolutional Neural Networks to Classify Alzheimer’s Disease Severity Based on SPECT Images: A Comparative Study
Source: J Clin Med. 2023 Mar 13;12(6):2218. doi: 10.3390/jcm12062218 (PMC10052955; doi:10.3390/jcm12062218)
Supplement: Supplementary file 1 [file jcm-12-02218-s001.zip › jcm-2211651-supplementary.pdf]

Supplementary Materials

Supplementary Methods

Two-stage experimental process

In the first stage of the experiment, the conditions for screening CNN architectures were information-security risks and embedded devices. First, the study results can be applied to embedded terminal and small mobile devices in hospitals and can be integrated with the medical-diagnosis process. Thus, the development of artificial intelligence (AI) in the medical field can be promoted to improve diagnosis efficiency. However, because of the limited storage space and power consumption of general devices, the feasibility of applying deep CNNs to embedded devices is low. Embedded terminal devices should be small and fast for maintaining detection accuracy. Second, with the rapid development of cloud services and IoT, non-information-related industries, such as medical and health information, have become increasingly dependent on information systems and networks, which has resulted in constant risks and threats to information security. Therefore, the protection of information security and the cost of network transmission cannot be ignored. In the early stage of the experiment, two lightweight CNNs (MobileNet V2 and NASNetMobile) were first used for image recognition. After repeated training, the proper adjustment of the model parameters had negligible effect on model accuracy. Finally, a method that can improve the recognition performance of lightweight CNNs through transfer learning is required. Considering that the data used in this study are

medical images that are highly complex, the nature of the images differs from that of general images. The details of the two light-weight CNN models are presented below.

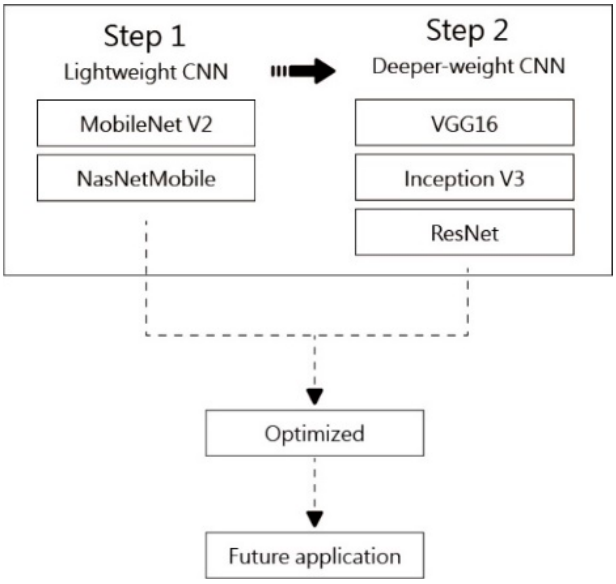

Figure S1. Two-stage experimental process used in this study.

MobileNetV2 [1]

Fine-tuning

To prevent excessive model parameters from causing overfitting problems, GlobalAveragePooling2D was added at the end of the model to replace the fully connected layer, and the average value of each feature map was considered to be the output. Next, two layers of batch normalization were added to ensure that the input of each layer of the network had the same distribution, which not only reduced the occurrence of overfitting but also accelerated the convergence of the training process. The output dimension of DenseNet was

set to 4,096. Finally, the dropout layer was added, and the ratio was set to 0.5 to improve model generalization. This result avoided reliance on certain regional features. Table 2 lists the layer settings at the end of MobileNetV2 after fine-tuning.

Experimental setup

Because MobileNetV2 was used the experimental model, the input image size of MobileNetV2 should be 224 × 224. Therefore, rescale = 1/255 was added in the preprocessing stage of the image data. Next, each pixel value of the original 124 × 120 SPECT image was multiplied by a scaling factor to facilitate model convergence. Simultaneously, the dataset was normalized to improve data integrity. The appearance and reading methods of all image data in all the records were ensured to be identical. To present the classification results as percentages, SoftMax was used as the model classifier. The ADAM optimizer was used to increase the training speed of the model. In the parameter part, the learning rate was set to 10^{-4}; the exponential decay rates of the first and second orders were 0.9 and 0.999, respectively, by default; epsilon was set to 10^{-8}; and the attenuation value was set to 10^{-5} using a low learning rate to retrain the new data. Categorical cross-entropy was used as the loss function, the batch size was set to 32, and the epoch number was set to 50. Insufficient sample data may lead to poor results after training and overfitting problems. To expand the dataset, the data enhancement method was used to horizontally flip, randomly zoom, and fill the original image (as shown in Table 2) to solve data imbalance and insufficient data problems.

NASNetMobile [2]

Fine-tuning

As described in MobileNetV2, to prevent overfitting caused by excessive model parameters and the limitation of the model-input size, GlobalAveragePooling2D was added at the end of the model to replace the final fully connected layer, and the average value of each feature map was used as the output to achieve dimensionality reduction. Simultaneously, feature information extracted from the previous convolutional and pooling layers was retained. The output dimension of the dense layer was set to 2048. Finally, a dropout layer was added, and the ratio was set to 0.5 to randomly disconnect a certain ratio of the input during the model training process and improve the generalization of the model to avoid relying excessively on certain regional features (as presented in Table 2).

Experimental setup

As described in MobileNetV2, rescale = 1/255 was added in the preprocessing stage of the image data to facilitate model convergence. To present the classification results as a percentage, SoftMax was added as the result classifier. Next, the stochastic gradient descent (SGD) was selected as the optimizer of the model to increase the training speed. In the parameter part, the learning rate was set to 10^{-4}, momentum was set to 0.9, and attenuation was set to 10^{-6}.

Categorical cross-entropy was used as the loss function, batch size was set to 32, and epoch number was set to 50.

In the second stage of the experiment, we used other deeper-weight CNN models for image recognition experiments to improve the nonlinear expression capabilities and learn complex features. Three deeper-weight CNN models (VGG16, Inception V3, and ResNet) were tested with SPECT images. The three types of CNNs were adjusted for the three influencing factors to optimize their performance. Comparative analysis can be performed to obtain the most suitable CNN architecture for SPECT images. The fine-tuning process of the fully connected layers in the deeper-weight CNN models in the second stage of this experiment is presented in Sections 2.4–2.6.

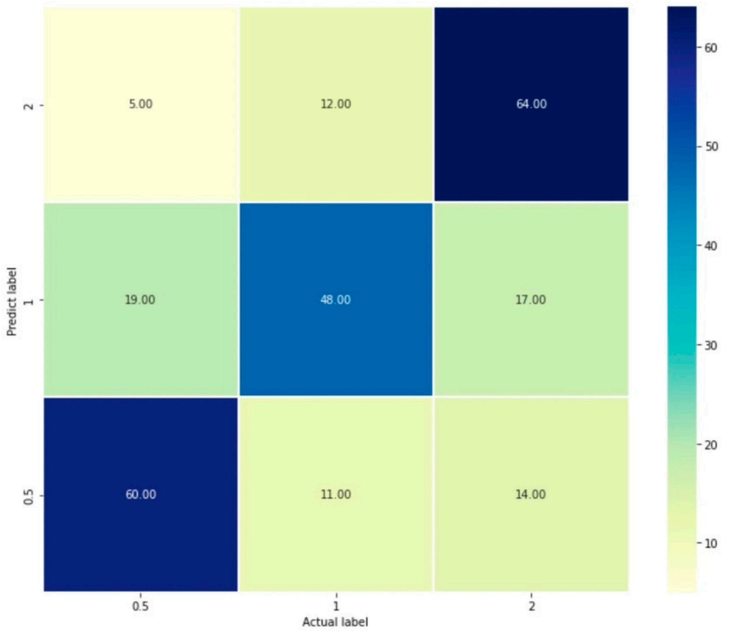

Figure S2. Confusion matrix of the ResNet model using the mixed test data of the SPECT brain images. 0.5: Clinical Dementia Rating Scale (CDR) = 0.5; 1: CDR = 1; 2: CDR = 2.

Table S1. Precision, recall, and F1 score of the ResNet model using the mixed test data of the SPECT brain images for each class of CDR scores.

	Precision	Recall	F1 score
CDR (0.5)	0.706	0.714	0.71
CDR (1)	0.571	0.676	0.619
CDR (2)	0.79	0.674	0.727

Abbreviations: CDR = Clinical Dementia Rating Scale.

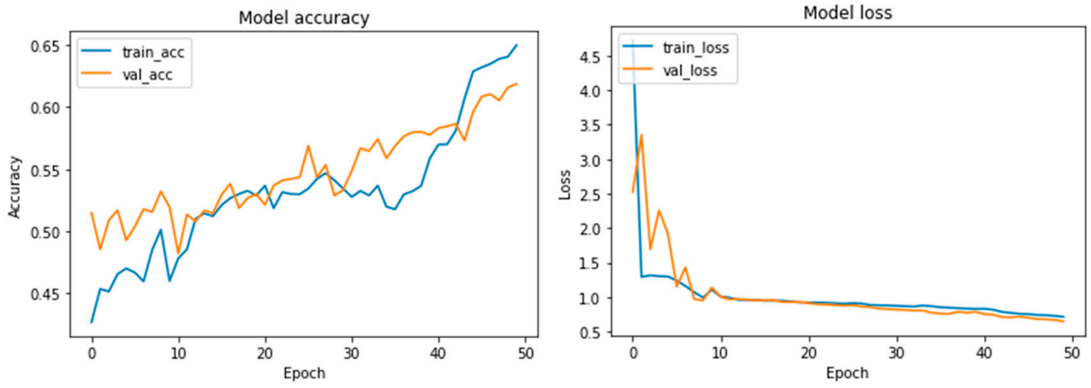

Figure S3. Learning curves of model accuracy and loss of mixed data using the MobileNetV2 model. The epoch of the MobileNetV2 model after transfer learning was set to 50, and the SPECT brain images were classified.

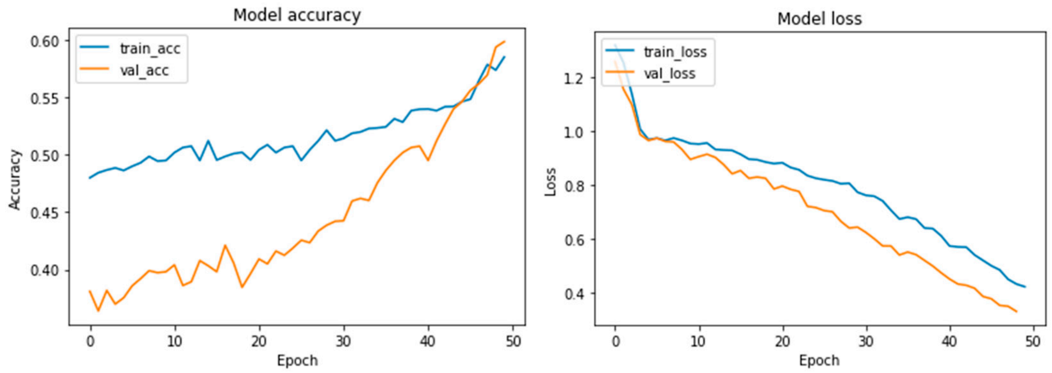

Figure S4. Learning curves of model accuracy and loss of mixed data using the NasNetMobile model. The epoch of the NasNetMobile model after transfer learning was set to 50 and SPECT brain images were classified.

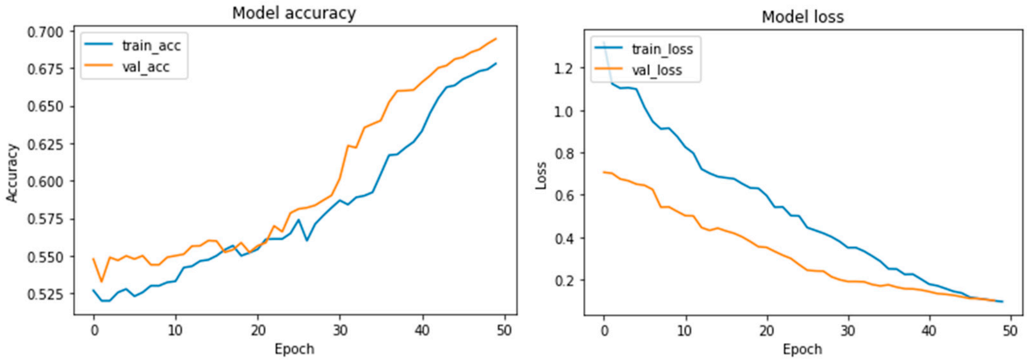

Figure S5. Learning curves of model accuracy and loss of mixed data using the VGG 16 model. The epoch of the VGG 16 model after transfer learning was set to 50 and the SPECT brain images were classified.

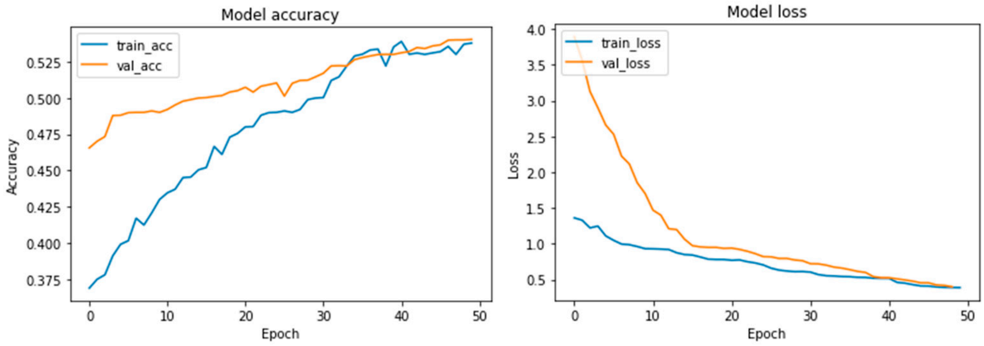

Figure S6. Learning curves of the model accuracy and loss of mixed data using the Inception V3 model. The epoch of the Inception V3 model after transfer learning was set to 50 and the SPECT brain images were classified.

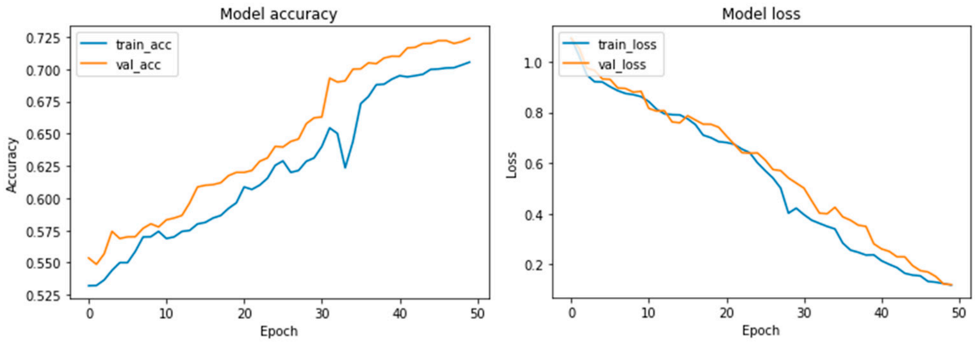

Figure S7. Learning curves of the model accuracy and loss of mixed data using the ResNet model. The epoch of the ResNet model after transfer learning was set to 50 and the SPECT brain images were classified.

References

[1] Sandler, M., Howard, A., Zhu, M., Zhmoginov, A., Chen, L. C. Mobilenetv2: Inverted residuals and linear bottlenecks. In Proceedings of the IEEE Conference on Computer Vision and Pattern Recognition, Salt Lake City, UT, USA, 18-23 June 2018, pp. 4510–4520.

[2] Zoph, B.; Vasudevan, V.; Shlens, J.; Le, Q.V. Learning transferable architectures for scalable image recognition. In Proceedings of the IEEE Conference on Computer Vision and Pattern Recognition, Salt Lake City, UT, USA, 18-23 June 2018, pp. 8697–8710.
